# Characterization of the complete chloroplast genome of *Toddalia asiatica* (L.) Lam. 

**DOI:** 10.1080/23802359.2021.1927220

**Published:** 2021-05-13

**Authors:** Pan Li, Kang Lei, Lusha Ji

**Affiliations:** School of Pharmacy, Liaocheng University, Liaocheng City, Shandong Province, PR China

**Keywords:** *Toddalia asiatica*, Rutaceae, phylogenetic, complete chloroplast genome

## Abstract

*Toddalia asiatica* (L.) Lam. belongs to family *Rutaceae* and mainly distributes in dry areas of bushes in tropical Africa, Asia, and Swaziland. Sometimes it can be used as fodder for goats, but it has been used as herbs in traditional medical treatment for 1000 years. In this study, we sequenced the sample of *T. asiatica* and determined its complete chloroplast genome. The length of CP genome is 158,434 bp, includes two invert repeats (IR) regions of 27,008 bp, a large single-copy (LSC) region of 86,132 bp, and a short single-copy (SSC) region of 18,286 bp. There are 133 genes, which includes 88 protein-coding genes, 8 rRNA and 37 tRNA, and 38.5% overall GC content. Each of *trn*K-UUU, *rps*16, *trn*G-UCC, *atp*F, *rpo*C1, *trn*L-UAA, *trn*V-UAC, *pet*B, *pet*D, *rpl*16, *rpl*2, *ndh*B, *trn*I-GAU, *trn*A-UGC, and *ndh*A genes contains a intron, *clp*P and *ycf*3 contains 2 intron. The phylogenetic analysis result shows that *T. asiatica* has the closest relationship with *Zanthoxylum armatum* (MT990984) and *Zanthoxylum nitidum* (MN508801).

*Toddalia asiatica* (L.) Lam. belongs to family *Rutaceae* and mainly distributes in dry areas of bushes in tropical Africa, Asia, and Swaziland (Duraipandiyan and Ignacimuthu 2009). Sometimes it can be used as fodder for goats, but it has been used as herbs in traditional medical treatment for 1000 years (Oketch-Rabah et al. 2000). The root and bark of *T. asiatica* have been used in traditional medicine to treat malaria, diarrhea, cholera, and cough, and the leaves can be used for lung and skin diseases (Ramaraj et al. 2012). The studies about functions of *T. asiatica* have been continued for long time, however, the information about its chloroplast genome could not be found in NCBI. In previous studies, they used DNA markers and different methods to constructed phylogenetic trees, and results showed that *T. asiatica* had close relationship with genus Zanthoxylum (Morton 2017; Appelhans et al. 2018;). In this study, we sequenced the complete chloroplast genome of *T. asiatica* and analyzed its phylogenetic relationship in *Rutaceae* by using complete chloroplast genomes.

The sample of *T. asiatica* was collected from South China Botanical Garden, Tianhe District, Guangzhou, Guangdong Province (N113°22′50″, E23°11′12″). We used the fresh leaves to extract chloroplast DNA-based CTAB method (Doyle and Doyle 1987) and construct the libraries with an average length of 350 bp using the NexteraXT DNA Library Preparation Kit (Illumina, San Diego, CA). Then the libraries were sequenced on Illumina Novaseq 6000 platform, over 2 Gb clean data was assembled with de novo assembler SPAdes version 3.11.0 software (Bankevich et al. 2012) and annotated by GeSeq (Tillich et al. 2017) with the chloroplast genome of *Zanthoxylum paniculatum* (MN968552) as reference. The raw data of sequence and annotation results were submitted to NCBI, under the accession number (MW194118) and SRA number SRR13479128. Furthermore, the sample was stored at Laboratory of Molecular Biology, Liaocheng University, Liaocheng (Voucher specimen: TA20200701LP) (Lusha Ji, Email: jilusha2020@163.com).

The complete chloroplast genome of *T. asiatica* is 158,434 bp in length and contains a large single-copy (LSC) of 86,132 bp, a small single-copy (SSC) of 18,286 bp, and two inverted repeat (IR) regions of 27,008 bp each. There were 133 genes, which includes 88 protein-coding genes, 8 rRNA, and 37 tRNA, and 38.5% overall GC content. Each of t*rn*K-UUU*, rps*16*, trn*G-UCC*, atpF, rpo*C1*, trn*L-UAA*, trn*V-UAC*, pet*B*, pet*D*, rpl*16*, rpl*2*, ndh*B*, trn*I-GAU*, trn*A-UGC, and *ndh*A genes contains a intron, *clp*P and *ycf*3 contains 2 intron。

To confirm the phylogenetic relationship of *T. asiatica* within *Rutaceae*. The complete chloroplast genome of 15 species in family *Rutaceae* was collected and aligned with *T. asiatica* by MAFFT version 7.037 (Katoh and Standley 2013). Subsequently, the phylogenetic tree was constructed by IQTREE version 1.6 (Nguyen et al. 2015; Hoang et al. 2018) with 1000 bootstraps replicates using Best-fit model. By using *Lagerstroemia villosa* (MK881633) as out group, we got the final ML tree, then Figure 1 showed that *T. asiatica* had the closest relationship with *Zanthoxylum armatum* (MT990984) and *Zanthoxylum nitidum* (MN508801). We got the similar result as previous studies. It could provide evidence for supporting the phylogenetic relationship between *T. asiatica* and genus *Zanthoxylum* within *Rutaceae* from complete chloroplast genome level.

**Figure 1.**
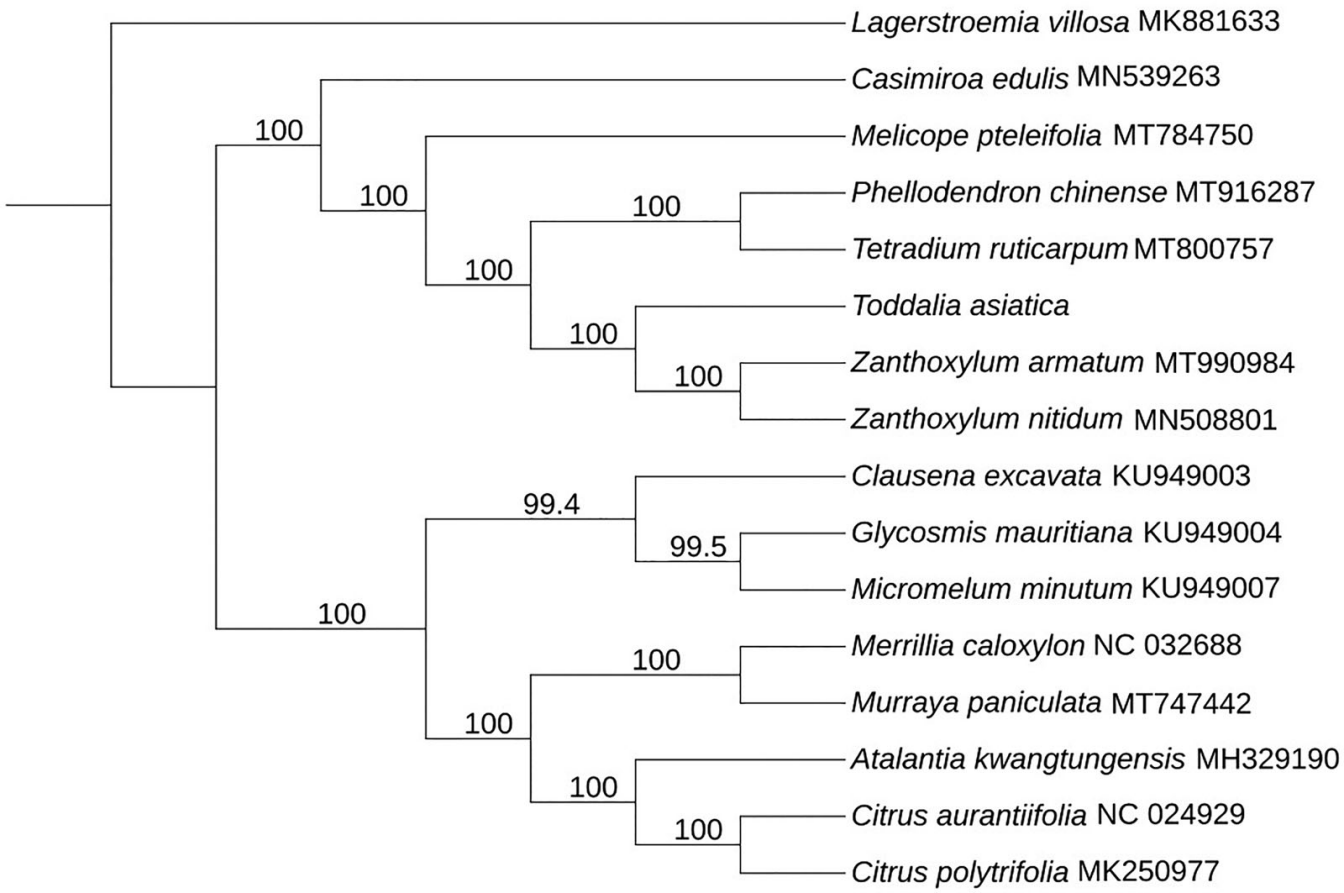
Maximum-likelihood phylogenetic tree for *T. asiatica* based on 16 complete chloroplast genomes.

## Data Availability

The genome sequence data that support the findings of this study are openly available in GenBank of NCBI at (https://www.ncbi.nlm.nih.gov/) under the accession no. MW194118. The associated BioProject, SRA, and Bio-Sample numbers are PRJNA693034, SRR13479128, and SRS8071277, respectively.
